# Bœnninghausen’s Aphorisms of Hippocrates—Toothache

**Published:** 1887-04

**Authors:** 


### BŒNNINGHAUSEN'S APHORISMS OF HIPPOCRATES.

### Toothache.

Translated by A. McNeil, M. D., San Francisco, Cal.

An instructive example of the selection of the homœopathic remedy is the following, which is an instance of the utility of an old theological *vs.* memorialis, in the treatment of a frequently returning toothache, as should be done by a homœopathic physician :

(Quis ?) Anna, a girl of some twenty years (Quid ?), complains of a violent toothache (Nebi ?) in a hollow, upper back tooth, on the left side, from which she has suffered a couple of months. In this general description there is not the remotest clue to the selection of the curative remedy, as more than half of all the proven drugs meet the conditions expressed. On further searching (Quibus auxiliis ?) for the concomitants of the patient we discover an anxious, timid, lachrymose disposition ; stomach easily disordered, particularly by fatty food ; disposition to mucous diarrhœa ; anxious palpitation of the heart in the evening when in the house ; falls asleep late ; evening chilliness, particularly in the back, with heat of the head and coldness of the extremities. However important and, in a certain measure, indispensable these symptoms are, yet the chief indications which are expressed in the above-mentioned verse are expressed by the words, *Cur ? Quomodo ? Quando ?* The *Cur ?* viz., refers to the often very important exciting cause or anamnesis, which in this case is stated to be a cold arising from wet feet, by which the menses, which were then flowing, were suppressed, and have not appeared since. The Quomodo ? however, refers to the nature of the pains, which are in this case twitching, tearing, and at times pulsating and stitching in the above-mentioned hollow tooth. They extend up the cheek to the eye, the temple, and the ear of that side. All the foregoing are less important than the final Quando ? which must have the aggravations and ameliorations according to time, attitude, or situation and circumstances, in order to make a certain and undoubted selection of the remedy. When, as in this case, the most painful period is in the evening till midnight, when the pains are aggravated when sitting quietly in a warm room, on becoming warm in bed, and especially by lying on the painless (not the painful)

side, and by hot or very warm food, and, on the contrary, are ameliorated in the morning and forenoon, when working in the open, cool air, and when cold water is held in the mouth the pains are considerably lessened or entirely cease.

Every homœopath knows that Pulsatilla and no other is the right remedy, which, administered in the smallest dose, not only removes with certainty the entire suffering, together with the concomitants, but with proper diet in the following days brings permanent cure.

This is the way, with the assistance and guidance of a sufficient familiarity with the homœopathic therapeutics, by which, in every kind of mental and physical complaints, the correct choice of the remedy can be reliably made. The physician is not thus misled into the dark region of supposition and hypothesis, where the scanty ray of light proves in the end an *ignis fatuus*. Such a procedure as ours may not demand any profound and astonishing *scientific knowledge*, but one may easily see that a rich and extensive experience, acquired by a wide knowledge, is indispensable to select from over one hundred remedies for toothache the only one which can cure, and that, too, in a disease that allopathy so seldom cures.

